# Size, Shape, and Distribution of Multivesicular Bodies in the Juvenile Rat Somatosensory Cortex: A 3D Electron Microscopy Study

**DOI:** 10.1093/cercor/bhz211

**Published:** 2019-10-29

**Authors:** M Turegano-Lopez, A Santuy, J DeFelipe, A Merchan-Perez

**Affiliations:** 1 Laboratorio Cajal de Circuitos Corticales, Centro de Tecnología Biomédica, Universidad Politécnica de Madrid, Pozuelo de Alarcón, 28223 Madrid, Spain; 2 Instituto Cajal, Consejo Superior de Investigaciones Científicas (CSIC), Avda Doctor Arce, 37, 28002 Madrid, Spain; 3 Centro de Investigación Biomédica en Red sobre Enfermedades Neurodegenerativas (CIBERNED) ISCIII, Madrid, Spain; 4 Departamento de Arquitectura y Tecnología de Sistemas Informáticos, Universidad Politécnica de Madrid, Pozuelo de Alarcón, 28223 Madrid, Spain

**Keywords:** cerebral cortex, endosomal pathway, multivesicular bodies, FIB-SEM, 3D electron microscopy

## Abstract

Multivesicular bodies (MVBs) are membrane-bound organelles that belong to the endosomal pathway. They participate in the transport, sorting, storage, recycling, degradation, and release of multiple substances. They interchange cargo with other organelles and participate in their renovation and degradation. We have used focused ion beam milling and scanning electron microscopy (FIB-SEM) to obtain stacks of serial sections from the neuropil of the somatosensory cortex of the juvenile rat. Using dedicated software, we have 3D-reconstructed 1618 MVBs. The mean density of MVBs was 0.21 per cubic micron. They were unequally distributed between dendrites (39.14%), axons (18.16%), and nonsynaptic cell processes (42.70%). About one out of five MVBs (18.16%) were docked on mitochondria, representing the process by which the endosomal pathway participates in mitochondrial maintenance. Other features of MVBs, such as the presence of tubular protrusions (6.66%) or clathrin coats (19.74%) can also be interpreted in functional terms, since both are typical of early endosomes. The sizes of MVBs follow a lognormal distribution, with differences across cortical layers and cellular compartments. The mean volume of dendritic MVBs is more than twice as large as the volume of axonic MVBs. In layer I, they are smaller, on average, than in the other layers.

## Introduction

Multivesicular bodies (MVBs) are membrane-bound organelles that contain intraluminal vesicles. MVBs were originally described in neurons (Palay and Palade 1955), but they are present in most cell types and tissues (Hanson and Cashikar 2012). They are involved in the transport, storage, sorting, recycling, and release of many substances (Von Bartheld and Altick 2011). MVBs interchange cargo with other organelles such as the Golgi complex, lysosomes, endoplasmic reticulum (Von Bartheld and Altick 2011), and mitochondria (Sugiura et al. 2014; Das et al. 2016). They also participate in autophagy and therefore in the degradation and repair of organelles, proteins, and RNA (Fader and Colombo 2009).

**Figure 1:**
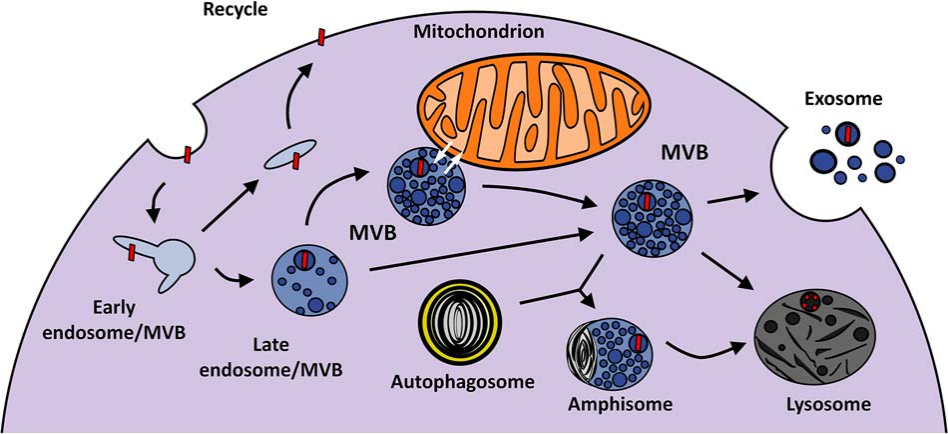
Schematic representation of the role of MVBs within the endosomal pathway. Endosomes originate by endocytosis, and intraluminal vesicles accumulate inside them as they mature. Cargo (red bars) transported by MVBs can come from the plasma membrane or from organelles such as mitochondria, as is the case in this example. The content of MVBs can be recycled, released to the extracellular space as exosomes, or degraded by fusion with lysosomes.

MVBs participate in the endosomal pathway, which begins with the formation of early endosomes by the fusion of endocytic vesicles coming from the plasma membrane. When endosomes accumulate intraluminal vesicles in their lumen, they become MVBs, although the two terms (endosome and MVB) are commonly used interchangeably (Huotari and Helenius 2011). Molecules to be transported by MVBs (endocytosed macromolecules and cell membrane proteins) are mostly marked by monoubiquitination or by tetraspanins (Piper and Katzmann 2007; MacDonald et al. 2015). The incorporation of molecules into intraluminal vesicles is mediated by the ESCRT complex (Hurley 2008; Wegner et al. 2011), and they are then sorted toward three possible routes: recycling, exocytosis, or degradation (Elkin et al. 2016) (Fig. 1).

Molecules to be recycled are located in tubular protrusions of MVBs; from there, they are transported back to the cell membrane or to the *trans*-Golgi network, where they are directed to other destinations (Sachse et al. 2002; Von Bartheld and Altick 2011; Lu and Hong 2014). Another possible route is exocytosis. In this pathway, MVBs fuse with the cell membrane, so intraluminal vesicles and their cargo are released to the extracellular medium as exosomes. Exosomes may contain proteins (ubiquitin, AMPA receptors), lipids, and miRNAs that participate in cell-to-cell communication (Chivet et al. 2012, 2013). The third possibility is the degradative pathway, involving the fusion of MVBs with lysosomes. Alternatively, MVBs can also fuse with autophagosomes, giving rise to hybrid organelles called amphisomes, which later fuse with lysosomes (Fader and Colombo 2009; Baixauli et al. 2014). The degradative pathway is particularly important for mitochondria, which generate oxidized molecules derived from their metabolism. Oxidized cargo is stored and transported in mitochondrial-derived vesicles that fuse with MVBs before being degraded in lysosomes (Sugiura et al. 2014).

Numerous studies have linked the endosomic pathway and synaptic physiology (Hiester et al. 2018). This pathway is involved in the trafficking of synaptic receptors between extrasynaptic membranes and synapses (Kneussel and Hausrat 2016) and provides the membrane components required for the growth and maintenance of dendritic spines (Park et al. 2006). There is a recruitment of endosomes in dendritic spines during long-term potentiation (Wang et al. 2008) and an increase in the number of these organelles in the thorny excrescences of hippocampal CA3 pyramidal cells after spatial memory training (Stewart et al. 2005).

MVBs play an essential role in the clearance of some protein aggregates produced in neurodegenerative diseases (Filimonenko et al. 2007). Furthermore, alterations of the endocytic pathway are related to diseases characterized by the accumulation of aberrant proteins including: Alzheimer’s disease (Takahashi et al. 2002; Friedrich et al. 2010; Yamazaki et al. 2010; Funk et al. 2011; Vingtdeux et al. 2012; Goetzl et al. 2015; Trousdale and Kim 2015; Yuyama et al. 2015; Janas et al. 2016), Parkinson’s disease (Kurashige et al. 2013; Schreij et al. 2016), and Huntington’s disease (Von Bartheld and Altick 2011).

In this work, we have used high-resolution three-dimensional imaging acquired by focused ion beam (FIB) milling and scanning electron microscopy (SEM). The FIB is used to remove thin layers of material from the sample surface (20 nm of thickness in the present work). Images are then acquired with the SEM from the freshly exposed surface. The milling-imaging cycle is automated, so long series of images are obtained that represent three-dimensional samples of tissue. We have reconstructed 1618 MVBs from stacks of images acquired from the neuropil (i.e., the fine network of axons, dendrites and glial processes that exist between cell bodies and blood vessels). We have analyzed the density, volume, and spatial distribution of MVBs across the cortical layers of the somatosensory cortex (hindlimb representation), as well as their location within axons or dendrites. This study was performed using Wistar rats on postnatal day 14 (P14), as part of the Blue Brain Project, which is focused on collecting and integrating anatomical, molecular, and physiological data (Markram et al. 2015).

## Material and Methods

### Tissue Preparation and Three-Dimensional Electron Microscopy

Three male Wistar rats were sacrificed on postnatal day 14. Animals were given a lethal intraperitoneal injection of sodium pentobarbital (40 mg/kg) and were intracardially perfused with 2% paraformaldehyde and 2.5% glutaraldehyde in 0.1 M phosphate buffer. All animals were handled in accordance with the guidelines for animal research set out in the European Community Directive 2010/63/EU, and all procedures were approved by the local ethics committee of the Consejo Superior de Investigaciones Científicas (CSIC).

The brains were extracted from the skull and prepared for electron microscopy as previously described (Merchán-Pérez et al. 2009). Three-dimensional brain tissue samples of the somatosensory cortex (hindlimb representation) were obtained using combined focused ion beam milling and scanning electron microscopy (FIB-SEM). We used a Neon40 EsB electron microscope (Carl Zeiss NTS GmbH, Oberkochen, Germany) equipped with a gallium FIB and a field emission SEM column. Using the FIB, the sample surface is milled, such that a thin layer of material (20-nm thick) is removed. After removing each layer, the milling process is paused, and the freshly exposed surface is imaged with the SEM, using the backscattered electron detector at 1.8 kV acceleration potential. The milling and imaging cycles are sequentially repeated through a fully automated procedure, thus obtaining a stack of serial images representing a three dimensional sample of the tissue (Merchán-Pérez et al. 2009). Twenty-nine different samples (stacks of images) of the neuropil of the six layers of the somatosensory cortex were obtained (3 samples of layer I, 4 of layer II, 10 of layer III, 5 of layer IV, 3 of layer V, and 4 of layer VI). These image stacks have been used previously for the study of the spatial distribution of synapses (Anton-Sanchez et al. 2014; Merchan-Perez et al. 2014) and the identification and quantification of the synaptic targets on dendritic spines and shafts (Santuy et al. 2018a), as well as in the study of the size and shape of synapses (Santuy et al. 2018b), and the distribution of mitochondria in the neuropil (Santuy et al. 2018c). Image resolution in the XY plane ranged from 3.7 to 4.5 nm/pixel. Resolution in the *Z*-axis (section thickness) was 20 nm, and image sizes were 2048 × 1536 pixels. The number of sections per stack ranged from 189 to 363 (mean 254.66; total 7385 sections). The alignment (registration) of serial micrographs was performed with Fiji, an image processing package based on ImageJ software (Schindelin et al. 2012; Rueden et al. 2017). All measurements were corrected for tissue shrinkage that occurs during osmication and plastic embedding of the vibratome sections containing the area of interest (Merchán-Pérez et al. 2009). To estimate the shrinkage in our samples, we photographed and measured the vibratome sections with Stereo Investigator (MBF Bioscience), both before and after they were processed for electron microscopy. The values after processing were divided by the values before processing to obtain the volume, area, and linear shrinkage factors (Oorschot et al. 1991), yielding correction factors of 0.73, 0.81, and 0.90, respectively. The volumes of the stacks, after correction for tissue shrinkage, ranged from 225.13 to 508.96 μm^3^ (mean 306.55 μm^3^; total 8889.82 μm^3^). To estimate the density of MVBs in each stack, we counted the number of MVBs within an unbiased three-dimensional counting frame of known volume (Howard and Reed 2004). The volumes of the counting frames ranged from 167.39 to 444.11 μm^3^ (mean 277.23 μm^3^, total 8039.65 μm^3^).

### Visualization and Identification of MVBs and Their Location

**Figure 2:**
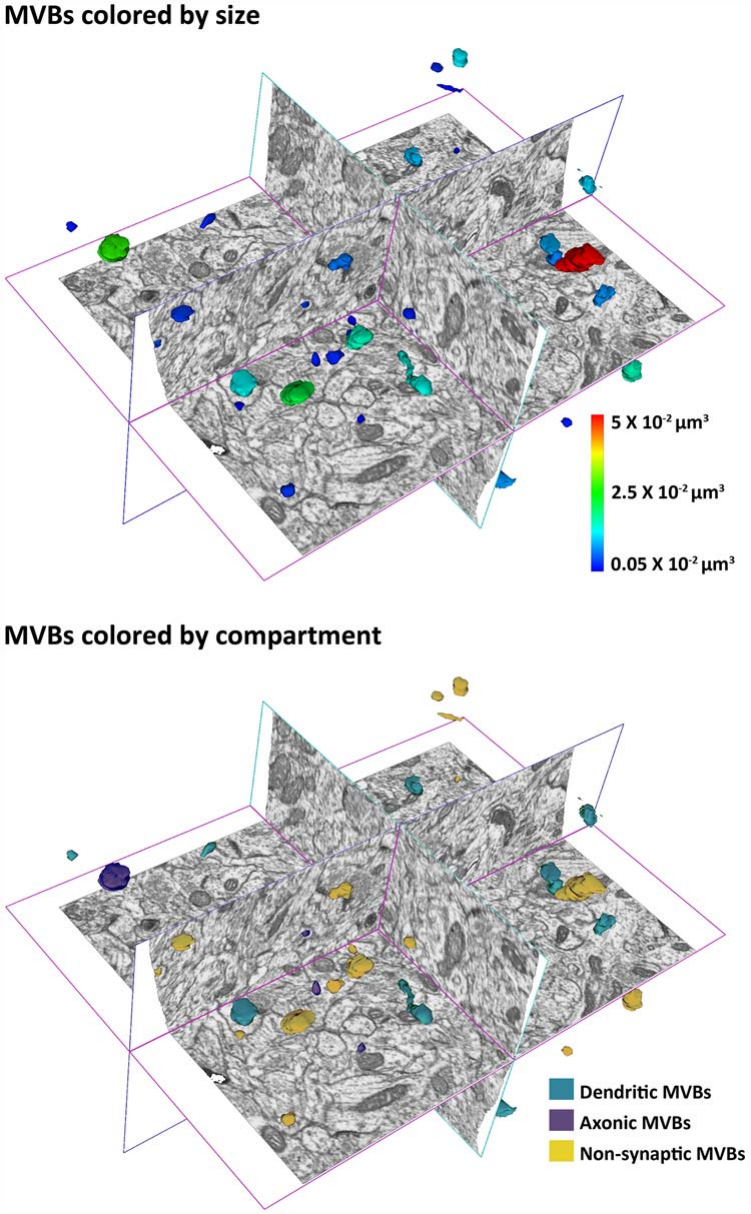
3D view of MVBs in a stack of images. MVBs are colored either by their size or by the compartment where they are located.

Single membrane organelles containing vesicles in their lumen were identified as MVBs, according to the original description by Palay and Palade (Palay and Palade 1955). MVBs were visualized and reconstructed in 3D with Espina software (Morales et al. 2011) (Fig. 2).

**Figure 3:**
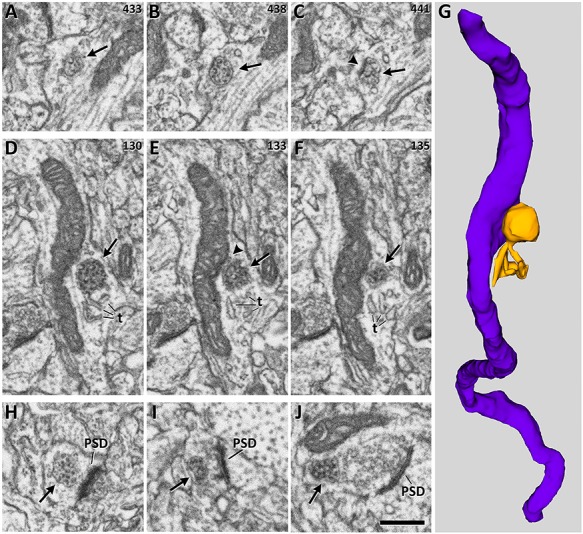
Examples of different types and locations of MVBs. *A*, *B*, *C*. Serial electron microscopy images showing a spheroidal MVB (arrows) that is isolated in the cytoplasm. Numbers in the top right-hand corner indicate the position of each micrograph in the series. Arrowhead in *C* points to a membrane thickening possibly representing a clathrin coat (see text for further explanation). *D*, *E*, *F*. Another series of images showing an MVB docked on a mitochondrion. The MVB (arrows) has several tubular expansions (t). Arrowhead in *E* shows the contact point between the mitochondrion and the MVB. *G*. Three-dimensional reconstruction of the same MVB and mitochondrion shown in *D*–*F*. *H*, *I*, *J*. MVBs located in a dendritic spine, an excitatory axon, and an inhibitory axon, respectively. Note that the postsynaptic density (PSD) is thick in excitatory synapses shown in *H* and *I*, while it is thin in the inhibitory synapse shown in *J*. Scale bar shown in *J* is 520 nm in *A* to *H*; 300 nm in *I*; 370 nm in *J*.

**Figure 4:**
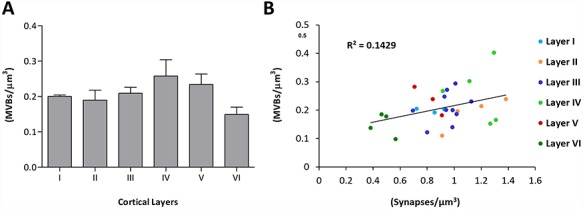
Density of MVBs in the neuropil. *A* Density of MVBs (MVBs/μm^3^) in the six cortical layers (mean + sem). *B* Scatterplot showing the lack of correlation between the density of MVBs and the density of synapses in the six cortical layers (*R*^2^ = 0.1429).

**Figure 5:**
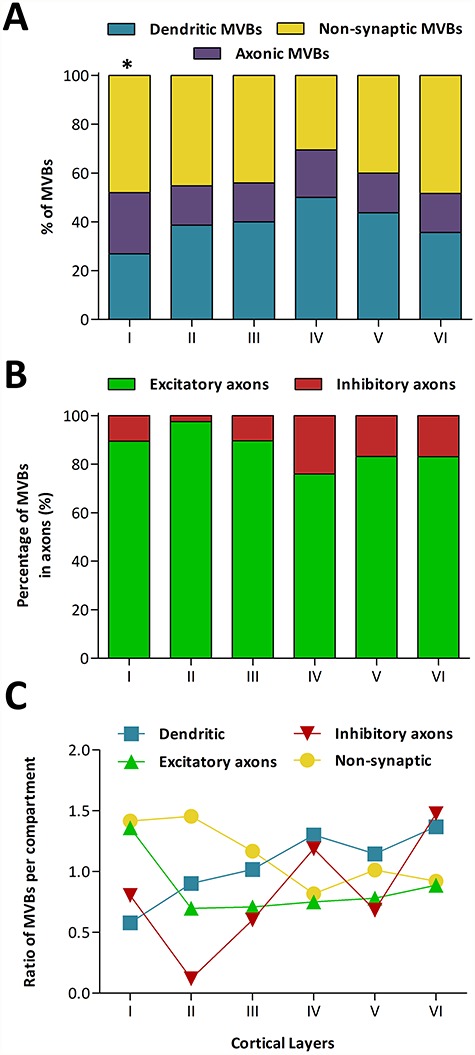
Distribution of MVBs across cortical layers and subcellular compartments. *A* Percentage of MVBs located in dendrites, axons, and nonsynaptic fibers across cortical layers. ^*^In layer I, the proportions of dendritic and axonic MVBs were lower and higher, respectively, than in any other layer (χ2, *P* < 0.05). *B* Percentage of MVBs located in excitatory and inhibitory axons. *C* Ratio between the percentage of MVBs in each compartment (nonsynaptic cell processes, dendrites, excitatory, and inhibitory axons) and the volume fraction occupied by each compartment. Values over 1 indicate that MVBs are relatively more concentrated in that compartment, while values below 1 indicate that MVBs are less concentrated.

**Figure 6:**
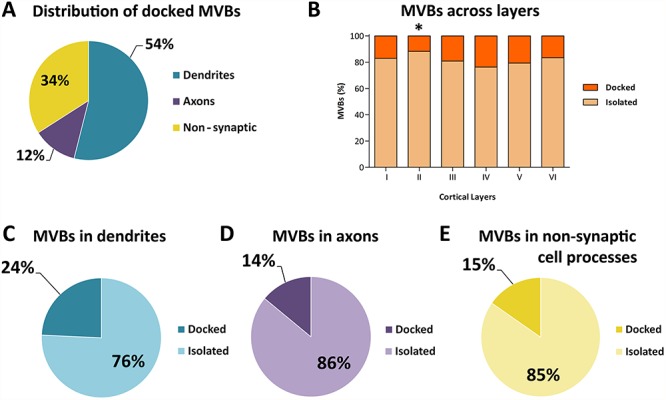
Distribution of MVBs docked on mitochondria and isolated in the cytoplasm. *A*. Distribution of MVBs docked on mitochondria in dendrites, axons, and nonsynaptic cell processes. *B*. Percentage of docked and isolated MVBs in the six cortical layers. ^*^The number of MVBs docked on mitochondria in layer II was lower than in any other layer (χ2, *P* < 0.05). *C*, *D*, *E*. Proportions of MVBs docked on mitochondria and isolated in the cytoplasm within the three subcellular compartments.

**Figure 7:**
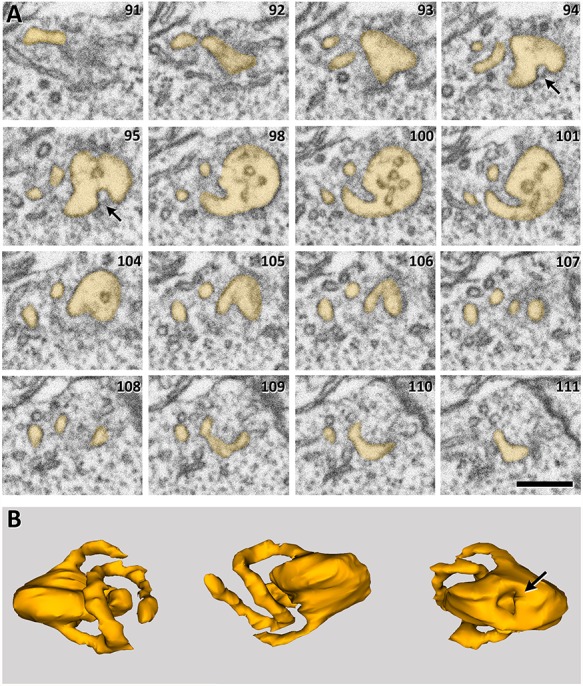
Example of an MVB with tubular protrusions. *A*. Serial images of an MVB (lightly stained) that shows tubular protrusions. An invagination of its membrane is also visible (arrows). The number in the top-right corner indicates the position of each micrograph in the series. Section thickness was 20 nm. Scale bar = 280 nm. *B*. 3D reconstruction of the MVB shown in *A*, from three different points of view. The arrow indicates the invagination of the membrane.

**Figure 8:**
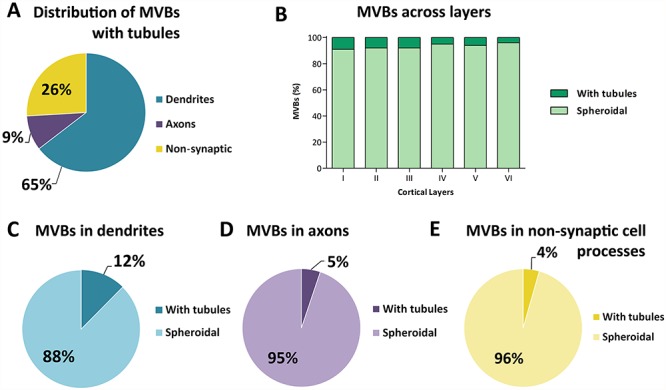
Distribution of spheroidal MVBs and MVBs with tubular protrusions. *A*. Distribution of MVBs with tubules in dendrites, axons, and nonsynaptic fibers. *B* Percentage of MVBs with tubules and without tubules (spheroidal) in the six cortical layers. *C*, *D*, *E*. Proportion of MVBs with and without tubules (spheroidal) in dendrites, axons, and nonsynaptic cell processes.

**Figure 9:**
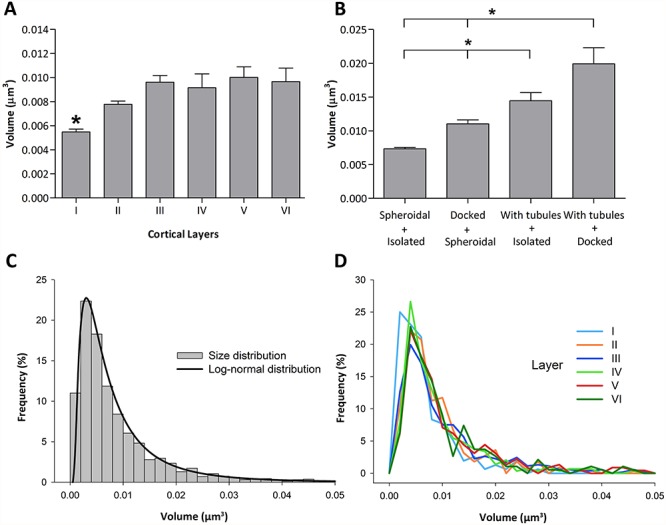
Size of MVBs across cortical layers. *A*. Size of MVBs in the six cortical layers (mean and standard error of the mean). ^*^The mean size of MVBs in layer I was smaller than in the other cortical layers (KW, *P* < 0.0001). *B*. Size of the different morphological types of MVBs. ^*^Differences in size were statistically significant (KW, *P* < 0.05) except between the two groups of MVBs with tubules (docked on mitochondria and isolated). *C*. Frequency histogram of the size of MVBs and the corresponding best-fit log-normal distribution (μ = −5.1375; σ = 0.9173). *D*. Frequency distribution of the size of MVBs in each cortical layer.

**Figure 10:**
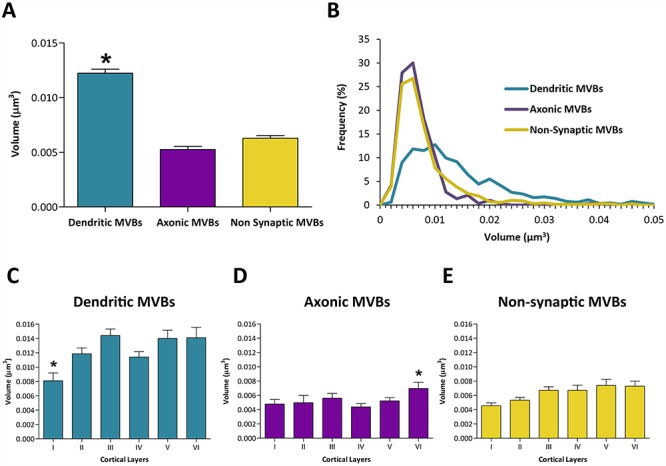
Size of MVBs in different subcellular compartments and cortical layers. *A*. Size of MVBs in different compartments (dendrites, axons, and nonsynaptic cell processes). ^*^The mean size of dendritic MVBs was larger than axonic and nonsynaptic MVBs (KW, *P* < 0.0001). *B*. Frequency histograms of the sizes of MVBs in the different compartments. *C*. Size of dendritic MVBs across cortical layers. ^*^Dendritic MVBs were smaller in layer I than in other layers except layer IV (KW, *P* < 0.05). *D*. Size of axonic MVBs across layers. ^*^Axonic MVBs in layer VI were larger than MVBs in layers I, II, and IV (KW, *P* < 0.05). *E*. Size of MVBs in nonsynaptic cell processes across the six cortical layers. Sizes are represented as mean volume (μm^3^) plus sem.

We measured the volume of each reconstructed MVB. In order to identify the subcellular compartment where they were located (axon, dendrite, or nonsynaptic process), we used Espina to navigate the stack of images in 3D (Santuy et al. 2018a, 2018b; Santuy et al. 2018a, 2018b). The subcellular compartments were classified as dendrites when they were postsynaptic or as axons when they were presynaptic with respect to at least one synaptic junction. Axons were further classified as excitatory or inhibitory when they established asymmetric or symmetric synapses, respectively (Gray 1959; Colonnier 1968). Myelinated fibers were classified as axons even if they did not establish synapses within the stack of images (Santuy et al. 2018a, 2018b). Any other cell processes that did not establish synapses were classified as “nonsynaptic.” The latter category would include glial processes but also some axonal or dendritic segments that did not establish synapses within the stacks of images (see Discussion). Given that the series of images were obtained from the neuropil, our samples did not contain cell somata or blood vessels.

The volume fraction occupied by MVBs in the neuropil of the six cortical layers was estimated using unbiased stereology. Briefly, a grid of 632 × 632 nm was superimposed on the images obtained by FIB-SEM (associated area of 400 000 nm^2^ per grid point). We then applied the Cavalieri’s principle, which states that the ratio between grid points hitting the object of interest and total points hitting the reference area is proportional to the volume fraction occupied by the object (Gundersen et al. 1988; Tang et al. 1997). For this purpose, we used a stereology toolbox from ImageJ (Mironov 2017). The estimations were made in every 40th section of each stack (*z* = 800 nm). The proportion of MVBs located in each compartment (axons, dendrites, and nonsynaptic processes) was compared with the volume fraction of those same compartments, estimated in a previous study on the same tissue (Santuy et al. 2018c).

### Spatial Distribution of MVBs

We used spatial statistics to analyze the distribution of MVBs in three-dimensional space. We used the spatial coordinates of the centers of gravity or centroids of MVBs to estimate the *G*, *F*, and *K* functions (Baddeley et al. 1993; Gaetan and Guyon 2010; O’Sullivan and Unwin 2014). We used the spatstat package of R software to estimate the three functions (Baddeley and Turner 2005). For this study, we used 21 stacks that had more than 25 points per sample. We tested the observed functions against an envelope generated by 100 simulations of the complete spatial randomness (CSR) model, also known as homogeneous spatial Poisson point process. The points of a given sample were considered to be distributed at random when all three functions lay within the envelope of the simulated CSR model. A similar approach has previously been used to study the spatial distribution of synapses (Blazquez-Llorca et al. 2013; Anton-Sanchez et al. 2014; Merchán-Pérez et al. 2014; Domínguez-Álvaro et al. 2018) and chandelier cells (Blazquez-Llorca et al. 2015).

### Statistical Analysis

Since normality and homoscedasticity criteria were not met, we performed nonparametric tests. We used the Mann–Whitney test (MW) and the Kruskal–Wallis test (KW) with Dunn’s posthoc. χ^2^ tests were used for contingency tables. Analyses were performed with Graphpad Prism 5 (Graphpad Software, Inc.). Curve fitting of probability density functions was performed with Easyfit Professional 5.5 (MathWave Technologies).

## Results

MVBs were visualized in the serial sections as single-membrane organelles containing vesicles in their lumen (Fig. 3). We identified and reconstructed 1618 complete MVBs in a total volume of 8039.65 μm^3^. Data were obtained from 29 stacks of images from the six cortical layers. The density of MVBs was calculated in each layer, and we determined the proportions of MVBs that were located within axons, dendrites, or nonsynaptic processes (Fig. 3*H*–*J*). The size and spatial distribution of MVBs were analyzed, as were other features such as their relationship with mitochondria, the presence of tubular protrusions, and the presence of clathrin coats (Fig. 3*C*–*G*). We found no statistically significant differences between animals regarding any of the parameters measured.

### Density of MVBs

The mean density of MVBs across layers was 0.21 ± 0.02 MVBs/μm^3^ (mean ± standard error of the mean [sem]). The maximum density was found in layer IV (0.26 ± 0.05) and the minimum in layer VI (0.15 ± 0.02) (Fig. 4*A*). Although the density varied between layers, these differences were not statistically significant (KW, *P* = 0.26). We compared the densities of MVBs and the densities of synapses that were obtained in a previous study from the same tissue samples (Santuy et al. 2018a) (Fig. 4*B*). It is interesting to note that layer IV presented both the highest densities of MVBs and synapses, while layer VI showed the lowest densities of MVBs and synapses. However, when we considered individual samples from all cortical layers, we did not find any correlation between the densities of MVBs and synapses (*R*^2^ = 0.14) (Fig. 4*B*).

### Location of MVBs in Dendrites, Axons, and NonSynaptic Cell Processes

We identified the cell processes containing MVBs by making use of the fact that the stacks of images could be visualized and navigated in 3D (see Material and Methods). Most MVBs (57.30%) were located in cell processes that established synapses, so they were classified as axonic or dendritic MVBs. The remaining MVBs were located in processes that did not establish synaptic connections, so they were tagged as nonsynaptic MVBs (42.70%) (Table 1, Fig. 5*A*).

**Table 1:** Percentages of MVBs located in dendrites, axons, and nonsynaptic fibers in the six layers of the cortex, and averaged for all layers (I–VI). Data given as percentage ± sem

Location of MVBs in different compartments in the six cortical layers (percent of MVBs ± sem)				
Layer	Dendrites	Excitatory axons	Inhibitory axons	Nonsynaptic cell processes
I	26.86 ± 2.86	22.50 ± 2.22	2.57 ± 0.33	48.07 ± 0.97
II	38.58 ± 4.73	15.78 ± 4.81	0.42 ± 0.42	45.22 ± 3.92
III	40.03 ± 2.63	13.98 ± 0.97	1.90 ± 0.65	44.09 ± 3.20
IV	50.04 ± 6.01	15.26 ± 3.86	4.22 ± 1.49	30.48 ± 3.10
V	43.71 ± 7.70	14.39 ± 4.54	1.90 ± 1.04	40.00 ± 4.06
VI	35.65 ± 5.91	13.31 ± 2.58	2.71 ± 1.07	48.33 ± 4.89
I–VI	39.14 ± 3.18	15.87 ± 1.37	2.29 ± 0.51	42.70 ± 2.74

In all layers, the percentage of MVBs located in dendrites was higher than the percentage of MVBs located in axons (Table 1, Fig. 5*A*). On average, across layers, 39.14% of MVBs were located in dendrites, while 18.15% were located in axons. In layers II to VI, dendritic MVBs clearly outnumbered axonic MVBs (Table 1). In layer I, the percentages of dendritic and axonic MVBs were very similar (26.86% and 25.07%, respectively). In fact, the proportion of dendritic MVBs in layer I was lower than in any other layer, while the proportion of axonic MVBs was higher than in any other layer (χ^2^, *P* < 0.05) (Table 1, Fig. 5*A*). Most dendritic MVBs (81.86%) were located in dendritic segments that presented at least one dendritic spine. The remaining dendritic MVBs (18.14%) were located in segments that did not show any spine in the volume of tissue analyzed.

We further classified axons containing MVBs as excitatory or inhibitory, based on whether they established asymmetric or symmetric synapses, respectively. We found that MVBs were more frequently found in excitatory axons than in inhibitory axons in all layers (average of 15.87% and 2.29%, respectively, Table 1, Fig. 5*B*). The differences between different layers were not statistically significant (χ^2^, *P* = 0.24). Regarding MVBs located in nonsynaptic processes, the highest percentage was found in layer VI (48.33%) and the lowest in layer IV (30.48%). Differences between layer IV and the rest of the layers were statistically significant (χ^2^, *P* < 0.05) (Table 1, Fig. 5*A*).

Differences in the proportions of MVBs located in dendrites, axons, and nonsynaptic processes could simply be due to the different volume fractions occupied by the three compartments. Alternatively, MVBs may be relatively more concentrated in some compartments and less concentrated in others. To explore these possibilities, we calculated the ratio between the proportion of MVBs within each compartment and the volume fraction occupied by that particular compartment (Fig. 5*C*), and we also performed a χ^2^ test comparing these proportions. The volume fractions of dendrites (38.50%), axons (22.81%), and nonsynaptic processes (38.69%) had been calculated previously for the same samples (Santuy et al. 2018c).

Our analysis indicates that MVBs were not homogeneously distributed between the three compartments (Fig. 5*C*). The most salient differences were found in layer I, where MVBs were relatively more concentrated in excitatory axons and nonsynaptic fibers, while they were less concentrated in dendrites and inhibitory axons (χ^2^, *P* < 0.001). Across layers, MVBs were relatively more concentrated in nonsynaptic cell processes in supragranular layers (χ^2^, *P* < 0.05) and less concentrated in layer IV (χ^2^, *P* < 0.05). Dendritic MVBs were relatively scarce in layers I and II, while they were concentrated in layer IV and infragranular layers (χ^2^, *P* < 0.05). Regarding excitatory axons, we found that MVBs were more concentrated in layer I, while the other layers had less MVBs than predicted based on the volume fraction of excitatory axons (χ^2^, *P* < 0.05). Finally, MVBs located within inhibitory axons showed the widest proportional variations, but this was probably due to the small number of MVBs (*n* = 35 in all layers) that were located in this type of axon (Fig. 5*C*).

### MVBs and Mitochondria

Most MVBs (81.84%) appeared as isolated organelles in the cytoplasm. There was, however, a large percentage of MVBs (18.16%) docked on mitochondria (Table 2, Fig. 6). Of the MVBs docked on mitochondria, most were found in dendrites (53.93%), followed by nonsynaptic cell processes (34.06%) and axons (12.01%) (Fig. 6*A*).

**Table 2:** Location of MVBs docked on mitochondria in dendrites, axons, and nonsynaptic cell processes in the six layers of the cortex, and averaged for all layers (I–VI). Data given as percentage ± sem

Location of MVBs docked on mitochondria in different subcellular compartments (percent of docked MVBs ± sem)			
Layer	Dendrites	Axons	Nonsynaptic cell processes
I	42.62 ± 10.10	13.09 ± 7.24	44.29 ± 2.97
II	59.44 ± 10.20	9.19 ± 5.95	31.37 ± 12.06
III	54.38 ± 7.48	10.40 ± 3.83	35.22 ± 7.37
IV	56.39 ± 2.72	17.77 ± 4.76	25.84 ± 6.60
V	55.82 ± 3.44	14.81 ± 14.81	29.37 ± 12.05
VI	54.92 ± 10.00	6.82 ± 6.82	38.26 ± 7.88
I–VI	53.93 ± 2.37	12.01 ± 1.63	34.06 ± 2.71

The percentages of MVBs docked on mitochondria varied between 23.69% in layer IV and 11.71% in layer II. The differences between cortical layers were not statistically significant, with the only exception being layer II (χ^2^, *P* < 0.05) (Fig. 6*B*).

Regarding the different subcellular compartments, we found different proportions of MVBs docked on mitochondria compared with those that were isolated in the cytoplasm. In dendrites, 24.25% of MVBs were docked on mitochondria, while the rest (75.75%) were isolated in the cytoplasm. In axons and nonsynaptic cell processes, the percentages of MVBs docked on mitochondria were found to be lower and similar (14.21% and 15.3%, respectively) (Fig. 6*C*,*D*,*E*).

Given that the amount of mitochondria varies across cortical layers (Santuy et al. 2018c), we examined whether there was any correlation between the numbers of MVBs and mitochondria (measured as their respective volume fractions). We found no correlation between the volume fractions of mitochondria and MVBs, regardless of whether the MVBs were isolated or docked (*R*^2^ < 0.2).

### MVBs with Tubular Protrusions

Most MVBs (93.34%) showed an irregularly spheroidal shape, and the remaining ones (6.66%) presented one or several tubular protrusions. Both types frequently presented invaginations in their membrane (Fig. 7). MVBs with tubules were found most frequently in dendrites (64.57%), followed by nonsynaptic fibers (25.93%) and axons (9.5%) (Fig. 8*A*; Supplementary Table S1).

MVBs with tubular protrusions were more frequently found in layer I (9.04% of all MVBs in this layer) and decreased toward layer VI (4.03%) (Fig. 8*B*). The differences between cortical layers were not statistically significant (χ^2^, *P* = 0.29).

The proportions of MVBs with tubules and spheroidal MVBs varied in different subcellular compartments (Fig. 8*C*,*D*,*E*). In dendrites, MVBs with tubular protrusions accounted for 12.39% of all dendritic MVBs, while they were found in lower and similar percentages in axons and nonsynaptic cell processes (5.17% and 4.42%, respectively).

### MVBs with Clathrin Coats

An electron-dense thickening was found in the membrane of 19.74% of MVBs (Fig. 3*A*, arrowhead). These thickenings have been previously described as clathrin coats, involved in protein sorting (Sachse et al. 2002; Klumperman and Raposo 2014). We observed that MVBs with clathrin coats were most frequently found in dendrites (58.77%) followed by nonsynaptic processes (28.15%) and axons (13.08%) (Supplementary Table S2).

In dendrites, the percentage of clathrin-coated MVBs was the highest (28.78%); it was lower in axons (16.88%) and lower still in nonsynaptic fibers (13.82%). In layers I and III, there were less clathrin-coated MVBs than in the other layers (χ^2^, *P* = 0.048).

The proportion of MVBs docked on mitochondria that had a clathrin coating was similar to that of the general population of MVBs (20.41% and 19.74%, respectively). However, in the case of MVBs with tubules, 43.69% of them presented a clathrin coat. The presence of a clathrin coat was unrelated to the volume of MVBs.

### The Size and Spatial Distribution of MVBs

We measured the size of MVBs reconstructed in 3D in all cortical layers. When all layers were considered together, we found that the mean volume of MVBs was 8.80 × 10^−3^ μm^3^, ranging from 5.57 × 10^−3^ μm^3^ in layer I to 9.77 × 10^−3^ μm^3^ in layer V (Table 3). The mean volume of MVBs in layer I was smaller than in the other cortical layers (KW, *P* < 0.0001), while differences between layers II to VI were not statistically significant (Fig. 9*A*). Similarly, we studied the size of the different morphological types of MVBs. We observed that MVBs with tubular protrusions that were docked on mitochondria had the largest mean volume (19.93 ± 2.38 × 10^−3^ μm^3^), followed by isolated MVBs with tubules (14.45 ± 1.21 × 10^−3^ μm^3^), spheroidal docked MVBs (11.03 ± 0.60 × 10^−3^ μm^3^), and finally, the smallest mean volume corresponded to spheroidal isolated MVBs (7.35 ± 0.20 × 10^−3^ μm^3^). These differences were statistically significant (KW, *P* < 0.05) except between the two groups of MVBs with tubules (docked on mitochondria and isolated) (Fig. 9*B*).

**Table 3:** Size of MVBs located in dendrites, axons, and nonsynaptic cell compartments in the six layers of the cortex and averaged for all layers (I–VI). Data given as μm^3^ × 10^−3^ ± sem

	Size of MVBs (μm^3^ × 10^−3^ ± sem) in different subcellular compartments			
Layer	All	Dendrites	Axons	Nonsynaptic cell processes
I	5.57 ± 0.41	8.09 ± 1.10	4.79 ± 0.65	4.55 ± 0.40
II	7.80 ± 0.45	11.86 ± 0.82	4.98 ± 1.02	5.32 ± 0.39
III	9.67 ± 0.47	14.42 ± 0.90	5.61 ± 0.68	6.71 ± 0.49
IV	8.48 ± 0.48	11.40 ± 0.78	4.38 ± 0.47	6.72 ± 0.72
V	9.77 ± 0.64	14.01 ± 1.14	5.23 ± 0.44	7.40 ± 0.87
VI	9.51 ± 0.66	14.12 ± 1.45	6.97 ± 0.86	7.28 ± 0.73
I–VI	8.80 ± 0.23	12.24 ± 0.36	5.25 ± 0.29	6.29 ± 0.24

To estimate the probability density function of MVB sizes, we plotted a frequency histogram and estimated the best fit curve (Fig. 9*C*). We found that MVB volumes followed a log-normal distribution with parameters μ = −5.1375 and σ = 0.9173. The volume of MVBs also followed log-normal distributions in the six cortical layers (Fig. 9*D* and Supplementary Fig. S1).

We compared the size of MVBs located in dendrites, axons, and nonsynaptic fibers (Table 3). The mean volume of dendritic MVBs was larger (12.24 × 10^−3^ μm^3^) than axonic (5.25 × 10^−3^ μm^3^) and nonsynaptic MVBs (6.29 × 10^−3^ μm^3^) (KW, *P* < 0.0001) (Table 3, Fig. 10*A*,*B*). The mean volume of MVBs located in excitatory axons (5.21 ± 0.31 × 10^−3^ μm^3^) was very similar to the volume of MVBs located in inhibitory axons (5.55 ± 0.81 × 10^−3^ μm^3^) (MW, *P* = 0.54).

We also explored the possible differences between cortical layers, in terms of the sizes of MVBs in dendrites, axons, and nonsynaptic cell processes. In dendrites, we only found statistically significant differences between the mean sizes of MVBs of layer I, that were smaller than the MVBs of other cortical layers except layer IV (KW, *P* < 0.05). In axons, MVBs of layer VI were, on average, larger than those of layers I, II, and IV (KW, *P* < 0.05). Finally, in nonsynaptic cell processes, there were no statistically significant differences in the size of MVBs across layers (Table 3, Fig. 10*C*,*D*,*E*).

When MVBs are reconstructed in 3D, Espina software records the coordinates of their centers of mass or centroids. We have used three spatial statistical tools (G, F, and K functions, see Material and Methods and Supplementary Fig. S2) to test the positions of MVBs in 21 stacks of serial sections. We found that in 12 of them, the MVBs were randomly distributed. The remaining samples, 9 out of 21, showed a slight tendency to cluster. Clustering was identified by the presence of empty spaces in the cloud of points (detected by the F function) and/or the distances to the nearest neighbor being shorter than would be expected by chance (detected by the G function) and/or local densities of points being higher than would be expected by chance (detected by the K function) (Supplementary Fig. S2).

## Discussion

One of the difficulties of studying MVBs is the lack of specific markers (Von Bartheld and Altick 2011). For this reason, electron microscopy is one of the best options to unambiguously identify and quantify MVBs accurately. Our method is based on automated three-dimensional electron microscopy using FIB-SEM, so relatively large volumes of tissue can be examined (more than 8000 μm^3^ in the present study), providing a good statistical sample to analyze the features and distribution of MVBs.

### MVBs and Synapses

Our study was performed in the neuropil, where the vast majority of cortical synapses are established (Alonso-Nanclares et al. 2008). We were therefore interested in the possible relationship between the numbers of synapses and MVBs in the different cortical layers. We observed that layers showing maximal and minimal densities of synapses (layers IV and VI, respectively) (Santuy et al. 2018a, 2018b) also, respectively, presented the highest and lowest densities of MVBs. However, when we considered individual samples from all cortical layers, we found no correlation between the number of synapses and MVBs. Therefore, although we cannot rule out the possibility that a subpopulation of MVBs is actually related to synapses, this relationship may be obscured by the presence of MVBs linked to other cellular functions. In fact, it has been described in hypoglossal neurons that MVBs labeled with BDNF or GDNF were commonly found close to synapses, while unlabeled MVBs and MVBs labeled with cardiotrophin or tetanus toxin were rarely close to postsynaptic densities (Rind et al. 2005). In addition, those MVBs that are related to synapses serve multiple dendritic spines simultaneously in the hippocampus (Cooney et al. 2002). Thus, further studies are necessary to determine the proportion of MVBs that are actually involved in synaptic physiology.

### Location in Subcellular Compartments

Previous studies have reported MVBs to be more abundant in dendrites and cell bodies than in axons (Altick et al. 2009; Von Bartheld and Altick 2011). In our samples, we found that MVBs are more frequent in dendrites than in axons with a ratio of slightly over 2:1. This is not only because dendrites occupy more space than axons, since the proportion of neuropil occupied by dendrites and axons is approximately 1.7:1 (Santuy et al. 2018a, 2018b). Therefore, we can conclude that dendrites are relatively enriched in MVBs when compared with axons. Also, dendritic MVBs are, on average, more than twice as large as axonic MVBs. Most dendritic MVBs (81.86%) were located in spiny dendritic segments, and the remaining 18.14% were found in dendritic segments that did not show any spine in the volume of tissue analyzed. This indicates that most dendritic MVBs would belong to spiny excitatory neurons, while a much smaller population would belong to aspiny inhibitory neurons. However, the exact percentages of MVBs located in dendrites of excitatory and inhibitory neurons cannot be accurately estimated from our present study. It should be considered, for example, that aspiny segments could belong to at least two different categories, in unknown proportions. First, aspiny segments may belong to truly aspiny inhibitory neurons. Second, some of these aspiny segments may belong to dendrites that do in fact have spines that are located outside the stacks of sections that we have analyzed. Therefore, the figure of 18.14% for MVBs located in aspiny dendrites most probably overestimates the actual percentage of MVBs in inhibitory dendrites.

Regarding excitatory and inhibitory axons, the proportion of MVBs in excitatory and inhibitory axons is approximately 7:1, while the respective volume fractions of these fibers is 6:1 (Santuy et al. 2018c), indicating that excitatory axons are relatively richer in MVBs than inhibitory axons. It is also important to note that MVBs located in nonsynaptic cell processes form the largest group (more than 40% on average). Many of the nonsynaptic cell processes that contain MVBs are probably glial processes. However, we cannot rule out the possibility that some of the processes classified as “nonsynaptic” are in fact axonal or dendritic segments that did not establish synapses within our tissue samples. Therefore, the percentage of MVBs in nonsynaptic cell processes should be considered as an upper limit.

The analysis of the distribution of MVBs across the different layers is much more complex. Layer I, for example, has approximately equal numbers of dendritic and axonic MVBs, while dendritic MVBs clearly predominate in every other layer. Moreover, MVBs in layer I have the smallest mean size of all layers. This could be explained by the distinctive composition of the neuropil of this layer. Since neuronal somata are very scarce, most dendrites will be distal, thinner dendrites originating from cell bodies located in deeper layers. It has been reported that mature endosomes tend to accumulate in the soma and proximal dendrites in cultures of neurons from rat and mouse embryos (Kulkarni and Maday 2018; Yap et al. 2018). Also, MVB size decreases with the distance to the cell nucleus in HeLa cells (Collinet et al. 2010). If this is also the case in our neocortical samples, this may explain the smaller proportion of MVBs in layer I dendrites, as well as their smaller volume. Although layer I shows the most marked differences, all the other layers also present variations in the numbers of MVBs located in axons, dendrites, and nonsynaptic cell processes. These differences may also depend on the particular cell composition of each layer and on the specific afferent and efferent projections. Another source of variability may come from the fact that the neocortex is not completely mature at this age (Jacobson 1963; Salami et al. 2003), so future studies would be required to determine possible maturational changes.

### Relation with Mitochondria

We have also found that MVBs are frequently in close contact with mitochondria. It has been reported that MVBs are involved in the transport and degradation of cargo to and from mitochondria (Sugiura et al. 2014; Das et al. 2016). On the one hand, the transport function has been shown by the transient “kiss and run” interaction between MVBs and mitochondria for the transference of iron-bound transferrin observed in erythroid and epithelial cells. On the other hand, the degradative function is represented by the fusion of MVBs and mitochondrial derived vesicles whose fate is degradation in the lysosome. It has also been recently described that MVBs carry mRNA along axons and associate with ribosomes, acting as local sites of protein synthesis. They often pause on mitochondria, where they act as platforms for the synthesis of proteins important for mitochondrial integrity and axon survival (Cioni et al. 2019). If the docking of MVBs to mitochondria is actually related to the synthesis, transport, and degradation of mitochondrial components, these activities would be intense in the neocortex, since almost every fifth MVB (18%) is docked on mitochondria. They are, however, unequally distributed among dendrites and axons, since MVBs docked on mitochondria in dendrites were 1.7 times more frequent than in axons, which may indicate a higher turnover of dendritic mitochondria.

### MVBs with Tubular Protrusions

Another morphological trait that may be related to a specific function of MVBs is the presence of tubular protrusions. MVBs with tubular protrusions are considered early endosomes, with activity related to sorting cargo, protein recycling, and transport toward the plasma membrane or the *trans*-Golgi network (Jovic et al. 2010; Huotari and Helenius 2011; Von Bartheld and Altick 2011). In our samples, approximately 7% of MVBs had tubular protrusions. Dendrites were again enriched in this kind of MVB. These results are in line with previous studies performed in cultures of dopaminergic and hippocampal neurons, which reported that early endosomes are mostly located in the somatodendritic compartment (Parton et al. 1992; Rao et al. 2011).

### MVBs with Clathrin Coats

MVBs sometimes present electron-dense regions of their membrane that have been identified as clathrin coats (Klumperman and Raposo 2014; Fermie et al. 2018). Clathrin coats are associated with the sorting and recruitment of cargo to be included in intraluminal vesicles, and they are essential for the communication between endosomes and the trans-Golgi network. This morphological feature is typical of the early and recycling endosomes (Sachse et al. 2002; Raiborg et al. 2006; Lu and Hong 2014). We found that 20% of the MVBs had clathrin coats, and dendritic MVBs had the highest number of clathrin-coated MVBs. Interestingly, approximately half of MVBs with tubules had a clathrin coat in their membranes, and both features (tubules and clathrin) are associated with early endosomes, with functions of sorting and recycling cargo (Klumperman and Raposo 2014).

### Size of MVBs

Our reconstructions in 3D provide valuable information about the size of MVBs. It has been reported that the size of MVBs is very variable (Altick et al. 2009; Von Bartheld and Altick 2011; Hanson and Cashikar 2012). We show, however, that this variability is constrained, since it consistently follows a log normal distribution. This distribution is characterized by a strongly skewed shape with a long tail to the right and is very common in many physiological and anatomical features (Buzsáki and Mizuseki 2014). Also, the size distribution of MVBs is continuous and no clear-cut boundaries can be found between different types of MVBs based on size alone. However, the mean size of MVBs does vary among the different cell compartments and cortical layers. Our results indicate that the mean volume of MVBs in dendrites is more than twice the mean volume of axonic MVBs, while the size of MVBs was similar in all cortical layers except in layer I where they were smaller, as mentioned above.

Variations in size have been linked to functional aspects of MVBs. For example, a previous study reported that in small, early MVBs there was no acid phosphatase activity, which would later appear with maturation (Altick et al. 2009). Different morphological types of MVBs have different mean sizes, although the size distributions greatly overlap. Further research is needed to ascertain whether these size differences can be attributed to functional and maturational aspects.

### Spatial Distribution

Finally, our analysis of the spatial distribution of MVBs reveals that they are randomly distributed, with a slight tendency to cluster in 43% of our samples. Although the possible significance of this observation is not known, this tendency toward clustering indicates that the distribution of MVBs in the neuropil is not totally homogenous, as suggested by the fact that they are more concentrated in the dendritic compartment.

### Concluding Remarks

In summary, MVBs are ubiquitous in all components of the neuropil of all cortical layers, indicating that house-keeping functions of the endosomal pathway are distributed along all kinds of neuronal and glial processes. We can also speculate that some functions are especially salient, as suggested by certain features of MVBs. For example, the frequent presence of clathrin coats and/or tubular extensions in MVBs suggests that recycling and sorting cargo are relatively important functions in the neuropil. Also, docking of MVBs on mitochondria is so common that mitochondrial maintenance seems to be a prominent activity.

However, care should be taken not to be too simplistic regarding the possible interpretation of the functional roles of MVBs based on morphological features. For example, clathrin coats, tubular extensions, and docking on mitochondria are not mutually exclusive features, suggesting that an individual MVB may have several functions whose complexity and dynamic changes cannot be elucidated with electron microcopy alone. On the contrary, other functions of the endosomal pathway, such as synaptic maintenance, may be underestimated in the present study simply because the MVBs responsible for such functions do not show any specific morphological trait.

In spite of being ubiquitous, MVBs are not homogeneously distributed across layers and cellular compartments. The fact that their distribution is not homogeneous may be due to multiple factors, including differences of cellular composition and inputs across cortical layers; differences in metabolic turnover between dendrites, axons, and glial processes; and maturational changes. These possibilities must be explored in the future in identified cell types and projections of known origin. To achieve this, the methodology that we have used here needs to be combined with cell labeling techniques and tract tracing methods. Other brain regions, ages, and species should also be studied, in order to identify common and diverging patterns of organization.

## Funding

The Spanish “Ministerio de Ciencia, Innovación y Universidades” (grant PGC2018-094307-B-I00 and the Cajal Blue Brain Project [C080020-09; the Spanish partner of the Blue Brain Project initiative from EPFL, Switzerland]; the European Union’s Horizon 2020 Research and Innovation Programme under grant agreement No. 785907 (Human Brain Project, SGA2) and; Centro de Investigacion en Red sobre Enfermedades Neurodegenerativas (CIBERNED, CB06/05/0066, Spain).

## Notes

The authors thank Carmen Alvarez, Miriam Marin, and Lorena Valdes for their technical assistance. *Conflict of Interest*: None declared.

## Supplementary Material

Supplementary_Material_Turegano-Lopez_et_al_bhz211
