# Supplementary material for: Size, Shape, and Distribution of Multivesicular Bodies in the Juvenile Rat Somatosensory Cortex: A 3D Electron Microscopy Study
Source: Cereb Cortex. 2019 Oct 29;30(3):1887–901. doi: 10.1093/cercor/bhz211 (PMC7132939; doi:10.1093/cercor/bhz211)
Supplement: Supplementary_Material_Turegano-Lopez_et_al_bhz211 [file supplementary_material_turegano-lopez_et_al_bhz211.pdf]

Supplementary Tables and Figures

Size, Shape and Distribution of Multivesicular Bodies in the Juvenile Rat Somatosensory Cortex: a 3D Electron Microscopy Study

M. Turégano-López, A. Santuy, J. DeFelipe, A. Merchan-Perez

Location of MVBs with tubular protrusions in different subcellular compartments (Percent of MVBs ± sem)			
Layer	Dendrites	Axons	Non-synaptic cell processes
I	41.12± 4.84	24.44± 12.37	34.44± 8.68
II	78.69± 8.55	7.14± 7.14	14.17± 9.47
III	69.31± 8.59	6.50 ± 4.48	24.19± 6.94
IV	67.78± 18.27	13.33± 6.67	18.89± 16.60
V	72.22± 14.70	5.56 ± 5.56	22.22± 14.70
VI	58.33± 25.00	0± 0.00	41.67± 25.00
I–VI	64.57± 5.42	9.50 ± 3.46	25.93± 4.18

Table S1. Location of MVBs with tubular protrusions. MVB location in dendrites, axons and non-synaptic cell processes in the six layers of the somatosensory cortex, and averaged for all layers (I–VI). Data given as percentage ± standard error of the mean (sem).

Location of MVBs with a coat of clathrin in different subcellular compartments (Percent of docked MVBs ± sem)			
Layer	Dendrites	Axons	Non-synaptic cell processes
I	30.76 ± 8.08	24.09 ± 2.15	45.15 ± 2.89
II	46.53 ± 14.73	18.40 ± 2.84	35.07 ± 9.27
III	58.48 ± 14.57	10.12 ± 2.59	31.40 ± 3.93
IV	67.06 ± 19.58	11.73 ± 5.11	21.21 ± 8.20
V	74.08 ± 21.03	4.76 ± 4.76	21.16 ± 7.45
VI	75.69 ± 20.46	9.38 ± 5.98	14.93 ± 5.65
I–VI	58.77 ± 7.13	13.08 ± 2.85	28.15 ± 4.54

Table S2. Location of MVBs with clathrin coat. MVB location in dendrites, axons and non-synaptic cell processes in the six layers of the somatosensory cortex, and averaged for all layers (I–VI). Data given as percentage ± standard error of the mean (sem).

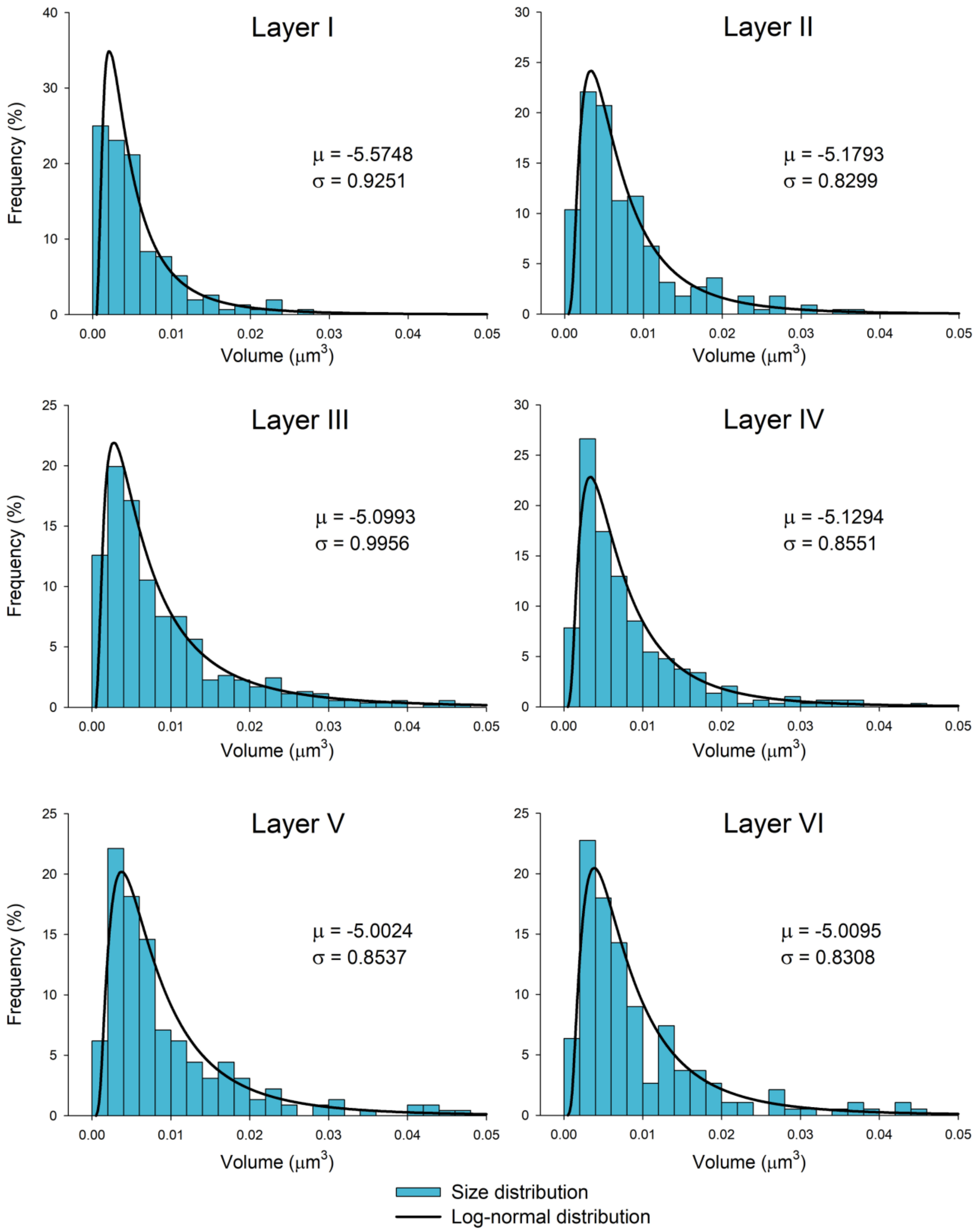

Fig S1. Frequency distribution of the size of MVBs in each cortical layer. In all layers, data fit a log normal distribution with the indicated μ and σ parameters.

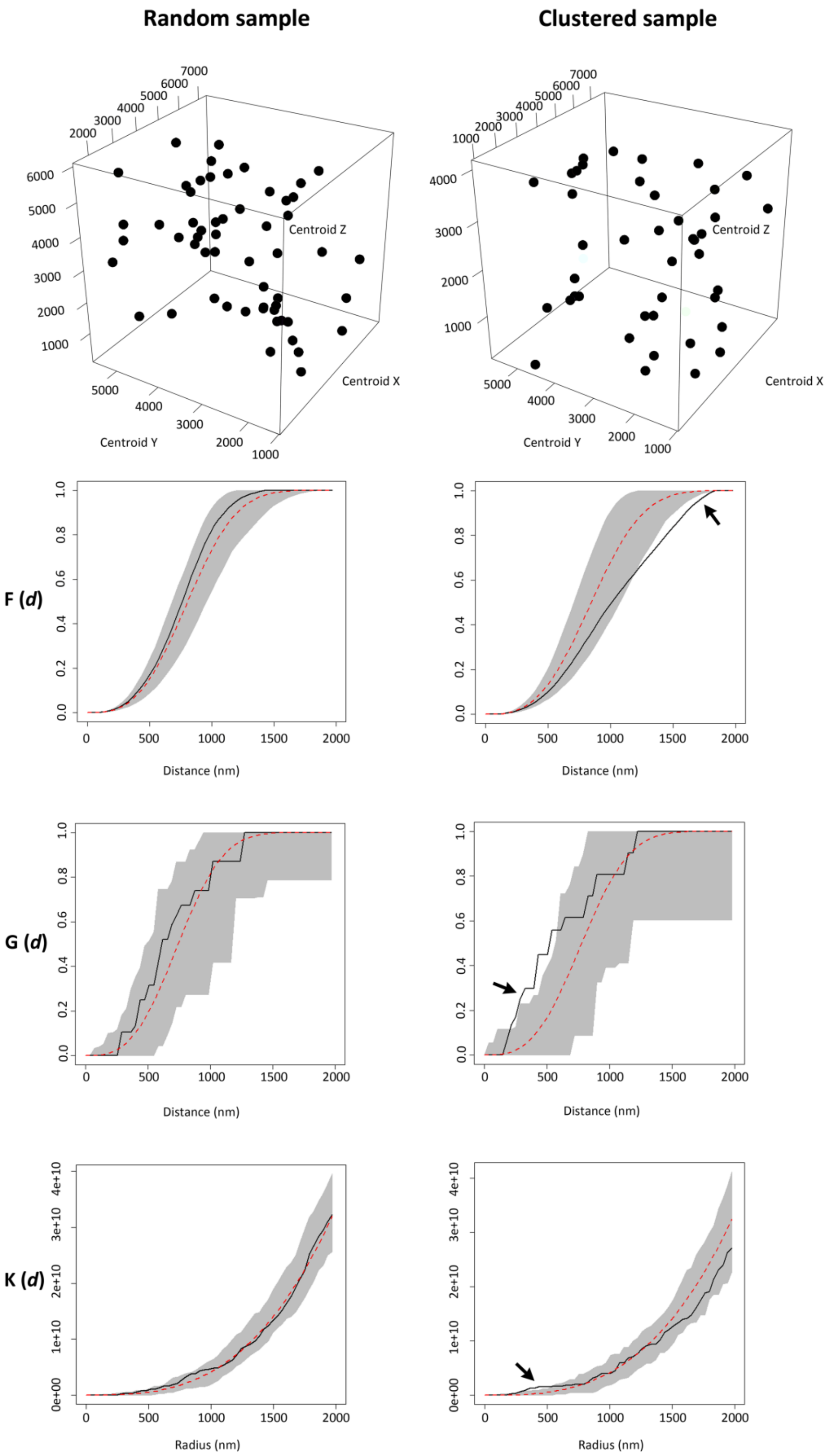

Fig S2. Spatial statistical analysis of the distribution of MVBs. Examples of randomly distributed MVBs (left column) and slightly clustered MVBs (right column). The top diagrams represent the spatial coordinates of MVBs in two stacks of serial sections obtained by FIB-SEM. For each of these samples, we have calculated the F, G and K functions (black lines). The red lines represent the theoretical complete spatial randomness or homogeneous Poisson point process. The grey envelopes have been generated by 100 simulations of a completely random distribution with the same number of points as the experimental sample. A sample is considered random only if the three functions lie within these envelopes, as in the left column. When the sample curve lies outside the envelope (arrows in the right column), the sample is not compatible with a random distribution. In this example, points are clustered, mainly because the distances to the nearest neighbor are slightly shorter than expected in a random distribution, as indicated by the G function.
